# Systemic inhibition of the membrane attack complex impedes neuroinflammation in chronic relapsing experimental autoimmune encephalomyelitis

**DOI:** 10.1186/s40478-018-0536-y

**Published:** 2018-05-03

**Authors:** Iliana Michailidou, Aldo Jongejan, Jeroen P. Vreijling, Theodosia Georgakopoulou, Marit B. de Wissel, Ruud A. Wolterman, Patrick Ruizendaal, Ngaisah Klar-Mohamad, Anita E. Grootemaat, Daisy I. Picavet, Vinod Kumar, Cees van Kooten, Trent M. Woodruff, B. Paul Morgan, Nicole N. van der Wel, Valeria Ramaglia, Kees Fluiter, Frank Baas

**Affiliations:** 1 Department of Genome Analysis, Academic Medical Center, Amsterdam, The Netherlands; 2 Department of Clinical Genetics, Leiden University Medical Center, Einthovenweg 20, 2333 ZC Leiden, The Netherlands; 3 Department of Bioinformatics, Academic Medical Center, Amsterdam, The Netherlands; 4 Department of Nephrology, Leiden University Medical Center, Leiden, The Netherlands; 5 Electron Microscopy Centre Amsterdam, Department of Medical Biology, Academic Medical Center, Amsterdam, The Netherlands; 6 School of Biomedical Sciences, The University of Queensland, Brisbane, Australia; 7 Systems Immunity University Research Institute, School of Medicine, Cardiff University, Cardiff, UK; 8 Department of Immunology, University of Toronto, Toronto, Canada

**Keywords:** Complement, Inflammasome, Neuroinflammation

## Abstract

The complement system is a key driver of neuroinflammation. Activation of complement by all pathways, results in the formation of the anaphylatoxin C5a and the membrane attack complex (MAC). Both initiate pro-inflammatory responses which can contribute to neurological disease. In this study, we delineate the specific roles of C5a receptor signaling and MAC formation during the progression of experimental autoimmune encephalomyelitis (EAE)-mediated neuroinflammation. MAC inhibition was achieved by subcutaneous administration of an antisense oligonucleotide specifically targeting murine *C6* mRNA (5 mg/kg). The C5a receptor 1 (C5aR1) was inhibited with the C5a receptor antagonist PMX205 (1.5 mg/kg). Both treatments were administered systemically and started after disease onset, at the symptomatic phase when lymphocytes are activated. We found that antisense-mediated knockdown of C6 expression outside the central nervous system prevented relapse of disease by impeding the activation of parenchymal neuroinflammatory responses, including the Nod-like receptor protein 3 (NLRP3) inflammasome. Furthermore, C6 antisense-mediated MAC inhibition protected from relapse-induced axonal and synaptic damage. In contrast, inhibition of C5aR1-mediated inflammation diminished expression of major pro-inflammatory mediators, but unlike C6 inhibition, it did not stop progression of neurological disability completely. Our study suggests that MAC is a key driver of neuroinflammation in this model, thereby MAC inhibition might be a relevant treatment for chronic neuroinflammatory diseases.

**Electronic supplementary material:**

The online version of this article (10.1186/s40478-018-0536-y) contains supplementary material, which is available to authorized users.

## Introduction

Neuroinflammation, or glial-cell propagated inflammation, is a broad concept describing the immune responses which are induced by inflammation or degeneration [43]. Initially considered as ‘bystander damage’ caused by cell injury, neuroinflammation was for a long time seen as a reaction to neuronal damage. This view was recently challenged in the light of evidence supporting a central role for neuroinflammation in neurodegenerative diseases. It is now known that the resident glial cells also have immunoprotective roles and can recruit peripheral immune cells into the central nervous system (CNS), if needed. Similar to most immune processes however, deviations from the delicate balance of homeostasis might prolong or exacerbate neuroinflammation, which in turn, promotes disease progression [14].

The complement system is a key component of innate immunity. Activation of the complement system involves various components, including C1q, mannan-binding lectin, C3 and C5, and has a critical role in the defense against infections, disposal of dead or dying cells, elimination of supernumerary synapses during development and response to ‘danger signals’ whether flagged by autoantibodies or recognized as altered-self. All pathways of complement activation lead to cleavage of the C5 molecule for generation of the anaphylatoxin C5a, and the opsonin C5b. C5b and the complement proteins C6 through C9, together form an oligomeric structure called the membrane attack complex (MAC) [66]. C5a and MAC have been shown as two major effectors of neuroinflammation and degeneration [4, 17, 47, 62, 63, 69, 85]. C5a exerts pronounced pro-inflammatory activity primarily through the G-coupled receptor for C5a (C5aR1) [81]. MAC causes cytolysis [66] or can act as an immune stimulating factor by promoting the secretion of interleukin 1 beta (IL-1β) via the Nod-like receptor protein 3 (NLRP3) inflammasome, when deposited at sub-lytic amounts [45, 78].

We propose that the terminal part of the complement system has a central role in neuroinflammation, driving degeneration. Because the individual roles of C5a and MAC in this process are not yet delineated we investigated the explicit effects of C5a and MAC on neuroinflammation using selective therapeutic inhibitors. Previous research on the role of MAC in neuroinflammation was performed under conditions in which MAC formation was inhibited already before induction of neuroinflammation [51, 83]. In these models one cannot rule out that induction of disease is affected by the deficiency of MAC. Here, we used the chronic relapsing experimental autoimmune encephalomyelitis (EAE) model of chronic neuroinflammation [35]. This model is used to study mechanisms of degeneration [44] and shows complement activation in the CNS [61]. Inhibition of the complement cascade was started after full induction of the disease, when T lymphocytes were activated. In order to discriminate between C5a- and MAC-mediated effects we selected the inhibitor PMX205, specific for the C5a receptor 1 (C5aR1), and the inhibitor RGS1104, a *C6* mRNA antisense oligonucleotide which blocks MAC formation. PMX205 is a C5aR1 antagonist with pharmacokinetics that allow inhibition in the CNS [85]. The C6 antisense oligonucleotide is an effective inhibitor of the *C6* mRNA, which is predominantly expressed in the liver [29]. Preventing production of C6 will effectively deplete the body thus, also prevent generation of MAC without affecting potential C5a-induced inflammation or upstream effects.

We show that systemic inhibition of MAC after the onset of disease prevented relapse completely in mice induced to develop chronic relapsing EAE whereas, inhibition of the C5aR1 mitigated neurological disability. Notably, MAC inhibition prevented the induction of major pro-inflammatory pathways within the mouse CNS, such as the NLRP3 inflammasome pathway. Histological examination of mouse spinal cords showed that MAC inhibition protected from relapse-induced axonal and synaptic damage or loss, two important pathological components of chronic relapsing EAE. Our data in mice suggest that MAC is a key contributor to neuroinflammation driving degeneration.

## Materials and methods

### Animals

Male 7 to 8-week-old Biozzi AB/H mice (*n* = 52) were obtained from Harlan (Bicester, U.K.) and housed individually in cages, at room temperature (RT), on a 12-h light: 12-h dark cycle. Animals weighed, on average, 26.93 ± 0.33 g at the beginning of the study. They were kept for at least 1 week before the start of the experiments. They were allowed free access to food and water for the entire duration of the study and provided with wetted mash as necessary. Animals were monitored for microbiological status according to the Federation of European Laboratory Animal Science Associations (FELASA) recommendations. All experiments were approved by the Academic Medical Center Animal Ethics Committee and complied with the Dutch national policy on humane care and the use of laboratory animals.

### Induction and clinical scoring of chronic relapsing EAE

Disease was induced in 8–12-week-old male Biozzi AB/H mice by subcutaneous injection into the flank, at both right and left sides, of 0.3 ml of a sonicated emulsion consisting of syngeneic Biozzi AB/H spinal cord homogenate (SCH) emulsified in complete Freund’s adjuvant (Sigma-Aldrich, St Louis, MO, USA), on days 0, 7, and of an emulsion consisting of SCH in incomplete Freund’s adjuvant (Sigma-Aldrich) on day 24, as previously described [5, 35]. Each animal received 1 mg of SCH and 60 μg mycobacteria [*Mycobacterium tuberculosis* H37Ra, *Mycobacterium butyricum* (4:1); Difco, BD Biosciences, San Jose, CA, USA] per injection [5, 35]. Body weight and clinical signs were assessed daily, as previously described [61], using the following five-point scoring system: 0, normal; 1, loss of tail tone; 2, impaired righting reflex; 3, partial hind limb paralysis, with 1 limb affected; 4, complete hind limb paralysis, with both limbs affected; and 5, moribund. Severity of clinical disability was further analyzed by quantitative (q) PCR analysis for selected immune genes (Additional file 1: Table S1). Remission from the active disease phase was defined as the resolution of clinical paralysis, weight gain and stabilization of the neurological deficit. Relapse was defined as an increase in clinical score of at least one point, development of paresis of the lower limbs and weight loss. Results are shown as the mean clinical scores ± standard error of the mean (SEM).

### Generation and administration of complement inhibitors

The C6 Locked Nucleic Acid (LNA) oligonucleotide (RGS1104, referred to as C6 antisense), blocker of the complement component C6, was synthesized with phosphorothioate backbones and methylated DNA-C (medC) by Ribotask (Odense, Denmark), on a Mermade 12, using 2 g NittoPhase (BioAutomation), as previously described [17]. Throughout the process, the oligonucleotide constitution was confirmed by MALDI-TOF mass spectrometry analysis on a Bruker Autoflex using 3-hydroxypicolinic acid as matrix. The sequence of the C6 antisense oligonucleotide is 5′-AACttgctgggAAT-3′ (LNA in capital letters, DNA in lowercase letters).

C6 antisense (5 mg/kg) was dissolved in phosphate buffered saline (PBS pH 7.4, Life technologies, Bleiswijk, The Netherlands) and delivered by osmotic pump at a rate of 0.25 μl per hour, over a period of 14 days (Alzet micro-osmotic pump, model 1002 Cupertino, CA, USA), starting on day 21 post immunization (p.i.). Phosphorothioate antisense oligonucleotides as used in these studies have a well-defined pharmacokinetic profile and biodistribution [21]. LNA wingmers like RGS1104 are efficiently taken up by the liver and mediate RNAseH-mediated mRNA cleavage. Subcutaneous dosing with an Alzet osmotic minipump at 5 mg/kg/day for 14 days reduced liver *C6* mRNA levels by 75%, whereas systemic C6 protein levels were reduced by 80%, as measured 10 days post-end of treatment (Additional file 2: Figure S1).

PMX205 [hydrocinnamate-(OPdChaWR)], blocker of C5aR1 [85], synthesized as previously described [47], was dissolved in distilled water and delivered by intraperitoneal injection (1.5 mg/kg), daily, starting on day 21 p.i., until the end of the experiment. The dose of PMX205 was sufficient to block C5aR1 signaling in the mouse CNS [7, 9]. A validated LC-MS/MS method was utilised for quantitative determination of PMX205 in plasma, brain and spinal cord tissue, using an API 3200 (AB SCIENX) mass spectrometer coupled with Agilent 1200 series liquid chromatographic system operated under multiple reaction mode [47]. The developed LC-MS/MS method has a limit of detection (LOD) and limit of quantification (LOQ) of 1.23 ng/ml and 3.73 ng/ml in plasma. LOD and LOQ in tissue is 1.95 ng/g and 3.28 ng/g.

### Experimental groups and tissue processing

Spinal cords from non-treated SCH-immunized Biozzi AB/H mice (referred to as no drug) were harvested for histology or RNA sequencing (RNA-seq) at four time points p.i. corresponding to: i) the initial acute paralytic attack (17 days p.i., *n* = 3), ii) the first remission (25 days p.i., *n* = 3), iii) the first relapse (38 days p.i., *n* = 7), and iv) the post-relapse phase (44 days p.i., *n* = 4). Spinal cords of SCH-immunized mice were compared to adjuvant-only (*n* = 9) or naïve (*n* = 3) controls.

To study the effect of C6 antisense on chronic relapsing EAE we collected spinal cords from mice immunized with SCH and treated with the C6 antisense oligonucleotide on day 38 p.i. (*n* = 7) and day 44 p.i. (*n* = 6). Similarly, to study the effect of PMX205 on chronic relapsing EAE we collected spinal cords from mice immunized with SCH and treated with the PMX205 drug on day 38 p.i. (*n* = 6) and day 44 p.i. (*n* = 4). Spinal cords from no drug, C6 antisense- and PMX205-treated animals were compared with each other, also compared with spinal cords from healthy control mice.

Mice whose spinal columns were harvested for RNA isolation and RNA-seq, were anesthetized and intracardially perfused with PBS. The spinal columns were excised and dissected into cervical, thoracic and lumbar regions, separately snap frozen. The frozen tissue was homogenized in TRIzol reagent (Invitrogen, Life Technologies, Carlsbad, CA, USA), and RNA was purified according to the manufacturer’s protocol. The concentration and quality of RNA were confirmed using a QuBit (Thermo Fisher Scientific, Waltham, MA USA) and a BioAnalyzer 2100 (Agilent, Santa Clara, CA, USA), respectively. All RNA RIN values were higher than 7.4.

Mice whose spinal columns were harvested for histology were anesthetized and intracardially perfused with PBS, followed by 4% paraformaldehyde. The spinal columns were excised and dissected into cervical, thoracic and lumbar regions; each region was further cut in two pieces and separately kept in formalin or glutaraldehyde for standard embedding in paraffin or epon, respectively.

### Elisa

Blood was collected from all mice (*n* = 52) in tubes coated with ethylenediaminetetraacetic acid (EDTA, Sarstedt, Nümbrecht, Germany) to measure serum levels of C6. The serum was separated by centrifugation twice at 5000 g for 10 min at 4 °C and the supernatant was pooled and stored at -80 °C. ELISA to detect C6 in the mouse serum was performed according to a standard published protocol [40].

### Quantitative (q) PCR

Liver was collected from all mice (*n* = 52) and immediately placed into RNA later, according to manufacturer’s instructions (Ambion, Bleiswijk, The Netherlands), to determine mRNA levels of *C6*. Total RNA was extracted from liver tissue using TriPure (Roche Nederland BV, Woerden, The Netherlands) and chloroform for phase separation and isopropanol precipitation. For the reverse transcription reaction, 0.5 μg RNA was mixed with 125 pmol/ml OligodT12-VN and denatured for 5 min at 65 °C. cDNA was synthesized using SuperScriptIII reverse transcriptase (Invitrogen) and incubating at 50 °C for 1 h. Quantification of RNA was done on a LightCycler 480 (Roche) with the Universal probe system (Roche). Hypoxanthine phosphoribosyl transferase was used as reference gene. The primers used are indicated in (Additional file 1: Table S2). qPCR was performed according to manufacturer’s instructions (Roche). Triplicates were used for each cDNA tested. Data were analyzed using the advanced relative quantification module in the LightCycler 480 software (Roche). qPCR was also performed on samples collected from homogenized spinal cords for quantification of expression levels of inflammasome or other immune genes (Additional file 1: Table S1; Additional file 2: Figure S2).

### RNA sequencing

Tissue was lysed in a MagNA Lyser using TriPure (Sigma-Aldrich) and MagNA Lyser green beads (Roche). RNA was isolated using Qiagen QIAcube and quality check was done with a BioAnalyzer RNA Nano Chip (Agilent). cDNA synthesis was performed using NuGEN Ovation RNA-Seq System V2 (7102-A01; NuGEN, San Carlos, CA, USA) followed by purification with Qiagen MinElute Kit. DNA was sheared to 200 to 400-bp fragments. The DNA was end polished and dA tailed, and adaptors with Bioo barcodes were ligated (Life Technologies). The fragments were amplified (eight cycles) and quantified with a QuBit (Thermo Fisher Scientific). Libraries were sequenced using the HiSeq PE cluster kit v4 (Illumina Inc., San Diego, CA, USA) on the HiSeq 2500 platform (Illumina), resulting in 125-bp reads.

### RNA sequencing analysis

Reads were trimmed using Trimmomatic [8] (v0.32) and subsequently aligned against the mm10 reference genome using HISAT2 [39] (v2.0.4) applying default parameters. Counts were obtained using the Ensembl GRCm38 GTF (v87) with HTSeq [1] (v0.6.1). Quality checks were performed with FastQC (https://www.bioinformatics.babraham.ac.uk/projects/fastqc/; v0.11.5). The data were analyzed using Ingenuity Pathway Analysis (IPA; QIAGEN Inc., https://www.qiagenbioinformatics.com/products/ingenuity-pathway-analysis/) [41].

### Light and fluorescence microscope

Six micron-thick paraffin sections were cut and collected on Superfrost Plus glass slides (VWR international, Leuven, Belgium), dried overnight at 37 °C and subsequently deparaffinized in xylene and rehydrated through a series of ethanol. Endogenous peroxidase activity was blocked by incubation in methanol (Merck, Darmstadt, Germany) with 0.3% H_2_O_2_ (Merck) for 20 min at RT. Sections were then rinsed in PBS and, where appropriate, pretreated with microwave antigen retrieval (3 min at 900 W followed by 10 min at 90 W) in either 0.05 M tris buffered saline (TBS, pH 7.6) or 10 mM citric acid buffer pH 6.0 or 10 mM Tris/1 mM ethylenediaminetetraacetic acid (EDTA) buffer pH 9.0 (Additional file 1: Table S3).

Following overnight incubation at 4 °C in the appropriate primary antibody (Additional file 1: Table S3) diluted in Normal Antibody Diluent (Immunologic, Duiven, The Netherlands), sections were incubated with a donkey anti-rabbit IgG HRP-linked antibody (diluted 1:1000 in PBS, Jackson ImmunoResearch Laboratories, Inc., West Grove, PA, USA) or with a donkey anti-goat IgG HRP-linked antibody (diluted 1:1000 in PBS, Jackson ImmunoResearch Laboratories) or with a donkey anti-mouse IgG HRP-linked antibody (diluted 1:1000 in PBS, Jackson ImmunoResearch Laboratories) or with the BrightVision poly-HRP-anti-rabbit IgG biotin-free (diluted 1:1 in PBS, Immunologic) for 45 min at RT. Incubation with the Vector® M.O.M.™ Immunodetection Kit (Vector Laboratories) was applied where appropriate. The immunostaining was visualized with 3,3′-diaminobenzidinetetrahydrochloride dihydrate (DAB, Vector Laboratories, Burlingame, CA, USA) or with the EnVision detection kit, containing a high sensitivity DAB chromogenic substrate system (Dako, Glostrup, Denmark). Sections were then counterstained with hematoxylin (Sigma-Aldrich) and mounted with VectaMount (Vector Laboratories).

To control for the specificity of the antibodies mouse spinal cord sections were stained according to the protocol described above except for the primary incubation step, which was omitted. In a second experiment, the primary antibody was subjected to an adsorption test using purified protein following an established protocol [55, 70]. In both cases, no signal was observed.

For fluorescent double immunostaining, primary antibodies (Additional file 1: Table S3) were incubated simultaneously overnight followed by incubation with the donkey anti-goat or mouse fluorescein isothiocyanate (FITC)-conjugated (Sigma-Aldrich) and the donkey anti-rabbit Cy3-conjugated secondary antibodies (Sigma-Aldrich) diluted 1:200 in PBS for 45 min at RT. Nuclear staining was visualized by 4′, 6-diamidino-2-phenylindole, (DAPI, Vector Laboratories) and sections were mounted in Vectashield (Vector Laboratories).

Images were acquired with a digital camera (DP25; Olympus, Zoeterwoude, The Netherlands) attached to a light microscope (Olympus, BX41, The Netherlands) for colorimetric staining or with a digital camera (DFC345FX, Olympus, The Netherlands) attached to a Leica-2 DM IRBE confocal microscope (Leica Microsystems BV, Rijswijk, The Netherlands) for immunofluorescent staining. Confocal image stacks were processed with Leica Application Suite (LAS) AF microscope software V2.3.5 (Leica Microsystems BV).

### Electron microscope

After the indicated treatment, the samples were fixed in Karnovsky’s glutaraldehyde (Polysciences, Inc., Warrington, PA, USA) and post-fixed with 1% osmium tetroxide (OsO4, Electron microscopy sciences, Hatfield, PA, USA; in cacodylate buffer). Subsequently, the samples were dehydrated in an alcohol series and embedded into Epon (LX-112 resin Ladd research, Williston, VT, USA). First semi-thin (1 μm) sections were cut for overview images and stained with Richardson’s staining solution [13]. Then ultrathin (80 nm) epon sections of the desired areas were cut and collected on Formvar-coated grids, counterstained with uranyl acetate and lead citrate. Sections were examined with a FEI Tecnai-12 G2 Spirit Biotwin electron microscope (Fei, Eindhoven, The Netherlands), and images were taken with a Veleta camera using Radius software (EMSIS, Münster, Germany).

### Quantification of immunoreactivity

Quantification of synaptophysin (SYP) and C9 reactivity was performed in the grey matter of stereotactic paraffin sections obtained from the cervical spinal cord segment. Quantification of Luxol fast blue (LFB) signal, proteolipid protein (PLP) reactivity or ionized calcium-binding adapter molecule 1 (IBA-1) reactivity was performed in paraffin sections obtained from the cervical or the thoracic spinal cord segment. Briefly, non-overlapping images covering > 95% of the area of interest were acquired from healthy control, no drug and C6 antisense-treated mice (8–10 images/section, 3 sections/mouse, 3–6 mice/group), at a 20× magnification using an Olympus BX41 microscope (Olympus), and processed with the Cell-D software (Olympus). Quantitative analysis of immunostaining was performed using the ‘measurement’ function of the Image J software (Image Pro Plus 5.1, National Institutes of Health, Bethesda, USA). Briefly, for each picture, the immunoreactive area was measured and divided to the total area of measurement. For measurement of the immunoreactive area a threshold was set and applied to all images in the same staining group. The percentage of immunoreactive area over the total area assessed was then calculated for each section. Average measurements per mouse were finally plotted for each group. Data were represented as mean ± SEM.

### Quantification of axonal damage

Quantification of axonal damage was performed in the ventrolateral column of Richardson-stained epoxy resin sections obtained from the cervical and thoracic spinal cord segments. Non-overlapping images covering > 95% of the ventrolateral column were acquired from the C4 and T4 level of healthy control, no drug and C6 antisense-treated mice (10–12 images/section, 2 sections/mouse, 4–6 mice/group). Images were captured at a 100× magnification using a Olympus BX41 microscope (Olympus). All animals were analyzed in a blinded fashion by two individual observers. Quantification of the damaged axons was performed using the Image J software (Image Pro Plus 5.1, National Institutes of Health, Bethesda, USA). Briefly, for each section an approximate number of 1000 axons was counted manually, using the ‘Cell Counter’ option of the ‘Plugins’ function. A second counter was then used for the quantification of damaged axons only. The percentage of damaged axons over the total number of counted axons was then calculated for each section. Damaged axons showed signs of demyelination and/or degeneration. Data were represented as mean ± SEM.

### Statistical analysis

Statistical tests were performed using Prism software (v7; GraphPad software, San Diego, CA, USA). The variable distribution was assessed by the Shapiro-Wilk test. When the test distribution was not normal, a non-parametric Kruskal–Wallis test was used followed by a Dunn’s multiple comparison test to assess intergroup differences. When the test distribution was normal, a One-Way Analysis of Variance (ANOVA) test was applied followed by Bonferroni correction for multiple comparisons. Data were represented as a mean ± SEM. The results were considered significant when *p* value < 0.05 at a 95% confidence level.

## Results

### Systemic inhibition of MAC or C5aR1 limits relapse

**Fig. 1:**
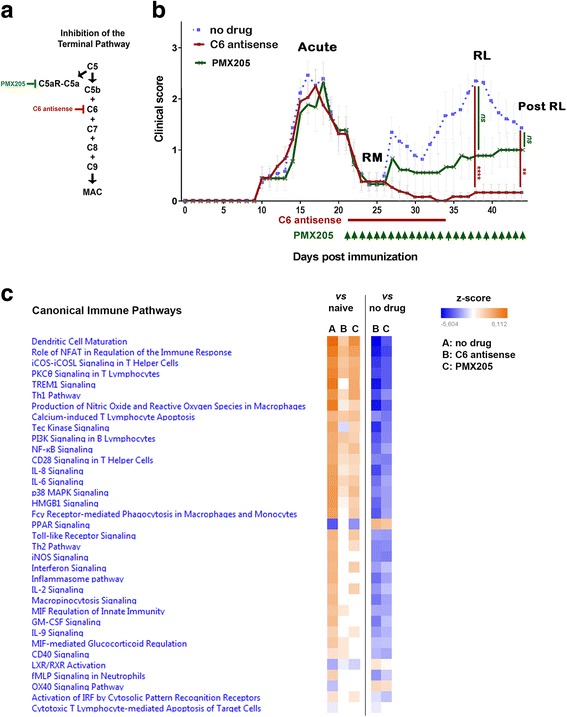
MAC inhibitor prevents relapse whereas, inhibitor of C5aR1-mediated inflammation ameliorates disability. Scheme illustrating inhibition of the terminal complement pathway **a**. at the level of C5b-C9 (MAC) by antisense targeting of complement *C6* mRNA (C6 antisense, 5 mg/kg), **b**. at the level of C5aR-mediated inflammation by an antagonist of C5aR1 (PMX205, 1.5 mg/kg) (**a**). Clinical scores of mice with chronic relapsing EAE receiving C6 antisense (*n* = 13, in red) or PMX205 (*n* = 10, in green) or no drug (*n* = 17, in blue). The C6 antisense-treated mice did not show relapse or neurological deterioration post-relapse phase. In contrast, PMX205 did not stop progression of neurological disability completely. Differences between groups were analyzed by using the Kruskal-Wallis test by ranks. Data represent the average clinical scores (mean) ± SEM. Statistical differences are indicated (**p < 0.01, ****p < 0.0001). RL, relapse; RM, remission; *ns*, not significant (**b**). Heatmap showing Ingenuity Pathway Analysis (IPA) canonical immune pathways induced by EAE and significantly affected by treatment with the C6 antisense or the PMX205 inhibitor. Pathways are ranked according to the z-score that predicts activation (orange) /suppression (blue). PMX205 is a less efficient inhibitor of neuroinflammation compared with the C6 antisense. Data were obtained from RNA-seq of mouse spinal cords collected at relapse (3 mice/group) (**c**)

To study the specific effect of MAC on neuroinflammation we used an antisense oligonucleotide (5 mg/kg) targeting the mRNA of complement component C6. The majority of C6 produced in the body is synthesized in the liver [29] and this oligonucleotide strongly reduces liver mRNA levels (Additional file 2: Figure S1). By reducing the *C6* mRNA levels in the liver the body is depleted of C6 (Additional file 2: Figure S1) and this prevents MAC assembly. We compared the effect of C6 antisense on clinical scores of mice with chronic relapsing EAE to that of PMX205 (1.5 mg/kg), an antagonist of C5aR1, thereby blocking C5aR1-mediated inflammation [47] (Fig. 1a). Unlike the C6 antisense oligonucleotide which cannot permeate the CNS, PMX205 enters the intact CNS (Additional file 2: Figure S3). Both drugs were systemically administered. Treatments started after disease onset, on day 21 p.i., between first disease episode and first relapse, when T lymphocytes were activated and disease-related damage was present.

Treatment with the C6 antisense oligonucleotide prevented relapse (clinical scores at day 38 p.i., C6 antisense vs no drug, 0.17 ± 0.16 vs 2.32 ± 0.34, mean ± SEM, ***p < 0.001) and all the C6 antisense-treated mice showed a stable phenotype post-relapse phase (clinical scores at day 44 p.i., C6 antisense vs no drug, 0.17 ± 0.16 vs 1.41 ± 0.33, mean ± SEM, **p < 0.01). Clinical scores in the PMX205-treated mice were lower but not significantly different compared to the no drug littermates (day 38 p.i., PMX205 vs no drug, 0.89 ± 0.35 vs 2.32 ± 0.34, mean ± SEM, *p* = 0.06; day 44 p.i., PMX205 vs no drug, 1.00 ± 0.33 vs 1.41 ± 0.33, mean ± SEM, *p* > 0.99; Fig. 1b).

To understand the mechanisms responsible for the C6 antisense- and PMX205-mediated effects on chronic relapsing EAE we analyzed the RNA expression profiles of spinal cords from C6 antisense-treated chronic relapsing EAE (*n* = 3) and PMX205-treated chronic relapsing EAE (*n* = 3) at relapse, at the point where there was the largest difference in clinical severity (day 38 p.i), and compared them with cords from no drug chronic relapsing EAE (*n* = 3) mice or cords from healthy controls (*n* = 3) to perform pathway analysis using IPA [41]. Focusing on canonical pathways we found that key inflammatory pathways were activated in the no drug mice when compared to healthy controls (colored orange, z-score > 0), while these pathways were partially activated or not altered (blank, z-score = 0) in the two treatment groups. Interestingly, the Tec kinase signaling was slightly downregulated in the C6 antisense-treated mice, but upregulated in the PMX205-treated mice. Comparison between each of the two treatments with the no drug mice showed a general downregulation of neuroinflammation-related pathways (colored blue, z-score < 0). Notably, PMX205 is a less efficient inhibitor of neuroinflammation compared with the C6 antisense. Moreover, two anti-inflammatory pathways, the peroxisome proliferator-activated receptor (PPAR) and the liver X receptor/retinoid X receptor (LXR/RXR) pathway, were downregulated in the no drug mice, but upregulated after treatment with the C6 antisense. Treatment with the PMX205 activated only the PPAR pathway, not the LXR/RXR pathway (Fig. 1c). This suggests that the treatments not only reduce inflammation but also specifically affect inflammation inhibitory pathways.

### Systemic MAC inhibition prevents upregulation of NLRP3 components in contrast to C5aR1 inhibition

Previous studies have shown that MAC activates the inflammasome [45, 78], initiating signaling pathways that drive inflammatory responses as shown for endothelial cells [38] and fibroblasts [80]. Activation of the inflammasome is critical for the induction of EAE since mice deficient in various inflammasome components are at least partially, resistant to the disease [19, 22, 28, 31–33, 36, 67, 74].

**Fig. 2:**
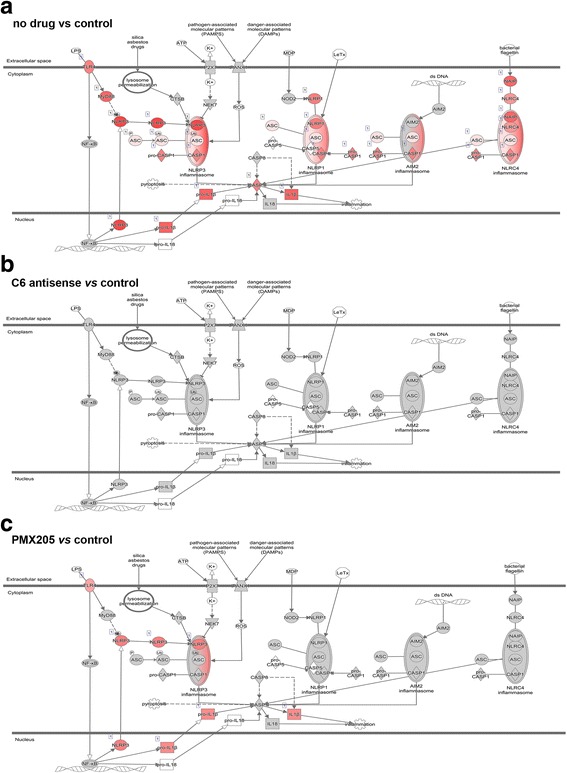
MAC inhibitor completely blocks NLRP3 inflammasome components while, in contrast, C5aR1 inhibition only partially decreases expression. The inflammasome pathway was modeled by Ingenuity Pathway Analysis (IPA) after analyses of gene expression data from mouse spinal cords collected at relapse (3 mice/group). Genes showing higher expression levels in the first compared to the second group of each comparison are colored red. The grey color indicates no changes of expression levels. Note that genes linked to NLRP3 inflammasome activation show no changes of expression levels in the C6 antisense group (MAC inhibitor, genes colored grey), but increased levels in the PMX205 group (inhibitor of C5aR1-mediated inflammation, genes colored red) when compared with healthy controls at relapse (**a-c**). Log fold change values of genes are shown in Additional file 1: Table S4

Analysis of RNA-seq data from spinal cords collected at relapse (day 38 p.i) showed that MAC-induced inflammasome activation occurred in chronic relapsing EAE. We found that the no drug mice showed upregulation of most genes related to the NLRP3 inflammasome pathway; in contrast, the C6 antisense-treated mice had no NLRP3 inflammasome gene expression. The PMX205-treated mice had reduced inflammasome gene expression compared to no drug mice, but still showed higher expression levels of genes linked to the NLRP3 inflammasome compared to the controls (Fig. 2a-c; for log fold change values of genes see Additional file 1: Table S4).

**Fig. 3:**
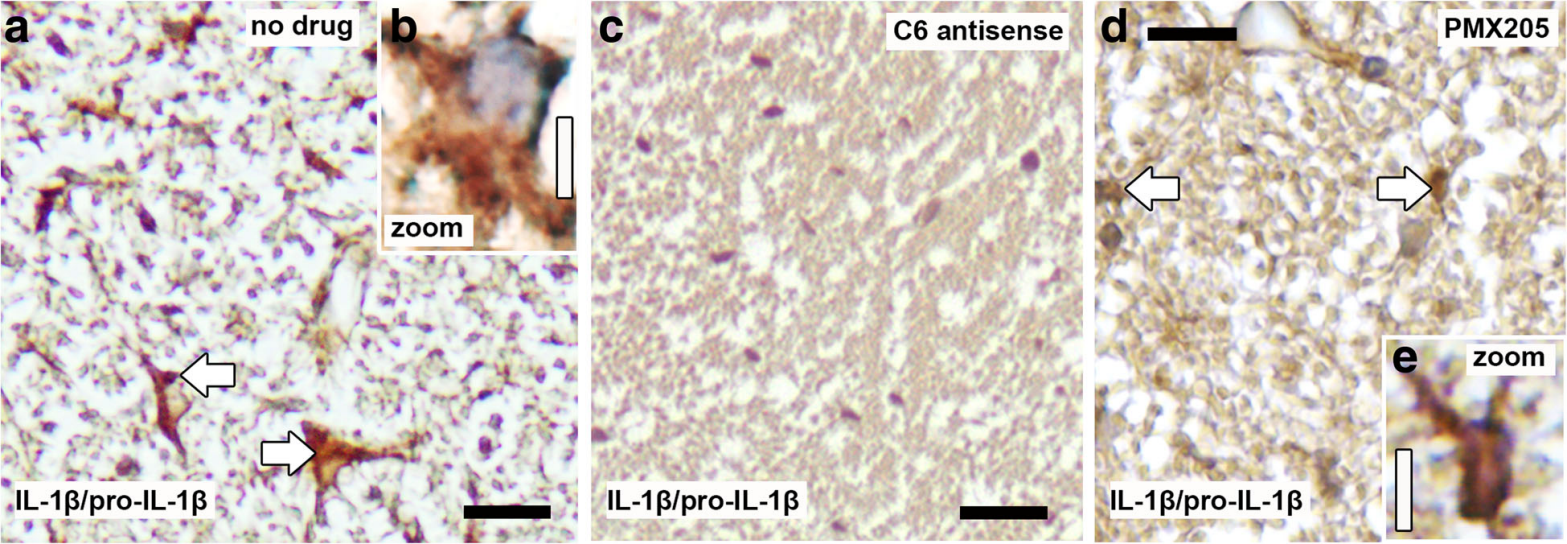
MAC inhibitor completely inhibits IL-1β synthesis while, in contrast, C5aR1 inhibition only partially decreases expression. Immunostaining for interleukin 1 beta (IL-1β)/pro-IL-1β, indicator of NLRP3 inflammasome activation, shows reactivity within the spinal cord of no drug mice (*n* = 4, arrows in **a**), on cells with a glial morphology (**a, b**). IL-1β/pro-IL-1β was never detected in the cords of C6 antisense-treated mice (*n* = 4) (**c**), while PMX205-treated mice showed sparse reactivity (*n* = 3) (arrows in **d, e**). Tissue was collected at relapse. Scale bars (**a**, **c**, **d**) 25 μm, (**b, e**) 5 μm. Hematoxylin was used as counterstain in (**a-e**)

Because inflammasome activation leads to cleavage of the precursor pro-IL-1β and release of the pro-inflammatory cytokine IL-1β [24] we performed immunostaining for IL-1β/pro-IL-1β on paraffin-embedded spinal cord tissue from C6 antisense-treated (*n* = 4), PMX205-treated (*n* = 3), and no drug (*n* = 4) mice, collected at relapse (day 38 p.i.). In line with our RNA-seq data, we found strong IL-1β/pro-IL-1β reactivity in the no drug mice, on glial cells, and sparse IL-1β/pro-IL-1β reactivity in the PMX205-treated mice. No IL-1β/pro-IL-1β immunoreactivity was ever observed in any of the C6 antisense-treated mice examined (Fig. 3a-e).

### Systemic MAC inhibition protects from axonal and synaptic damage

**Fig. 4:**
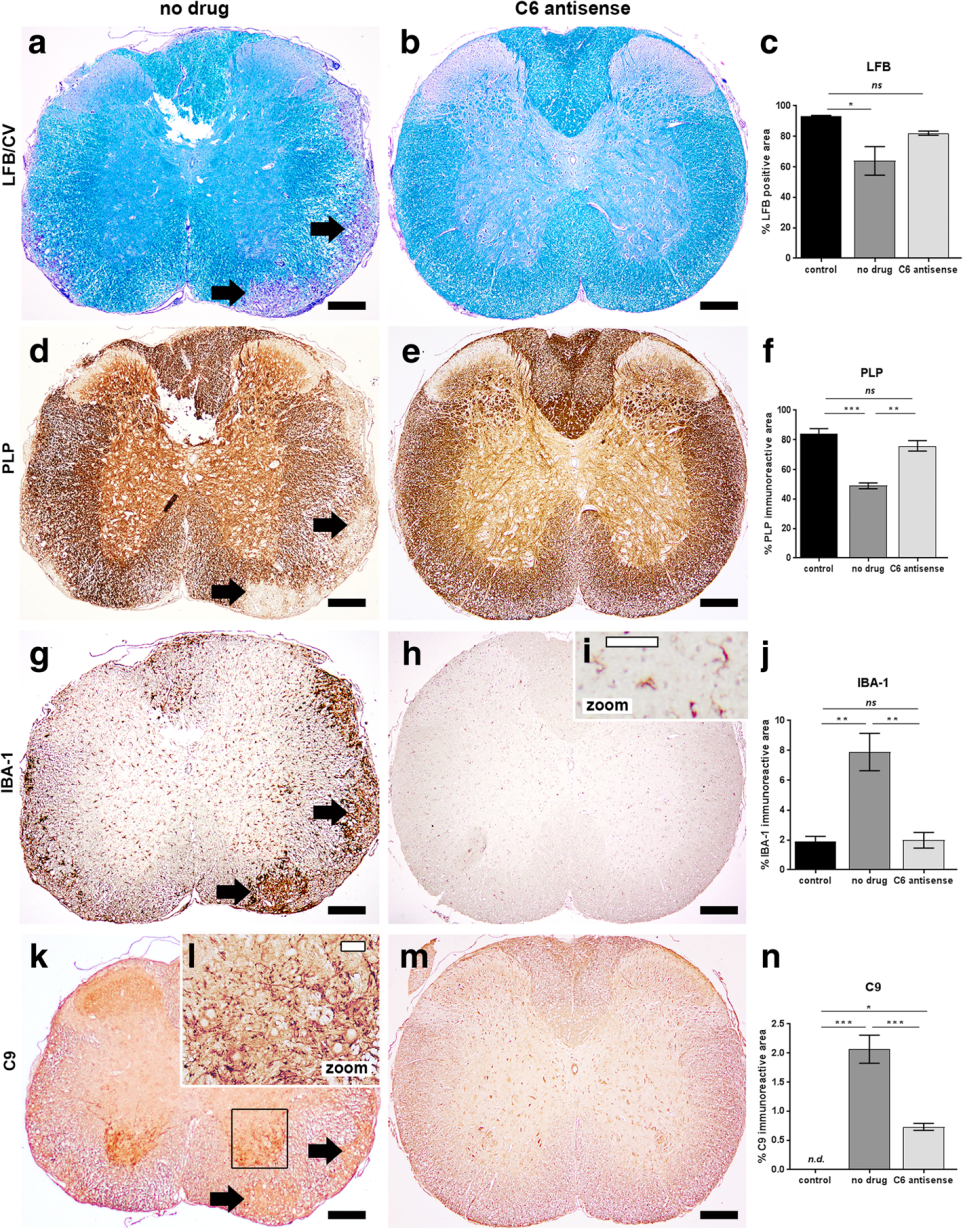
C6 antisense-mediated MAC inhibition prevents demyelination and microglia/macrophage activation in chronic relapsing EAE. Histological analysis of semi-serial paraffin sections of the mouse cervical spinal cord segment showed lesions in the no drug mice (*n* = 4) but no signs of active disease in the C6 antisense-treated mice (*n* = 6). Tissue was collected post-relapse phase. Staining for Luxol fast blue (LFB) and quantification of the signal (**a-c**) as well as staining for proteolipid protein (PLP) and quantification of the reactivity (**d-f**) indicated demyelination in the no drug mice (demyelinated lesions pointed by arrows in **a** and **d**), but no significant myelin loss in the C6 antisense-treated mice (**c**, **f**). Immunostaining for ionized calcium-binding adapter molecule 1 (IBA-1) (**g-j**) revealed microglia/macrophages within the lesions (arrows in **g**) and at the peri-lesional area of the no drug mice with a morphology consistent with an activated status. In contrast, all the C6 antisense-treated mice showed amounts of IBA-1+ reactivity similar to the ones found in controls and morphology of IBA-1+ microglia consistent with a resting status (**h**-**j**). Moreover, the no drug mice had abundant reactivity for C9, a marker of MAC deposition, within the ventral horns of the spinal cord. In contrast, the C6 antisense-treated mice showed only small amounts of MAC (**k**-**n**). Scale bars: (**a**, **b**, **d**, **e**, **g**, **h**, **k**, **m**) 200 μm, (**i, l**) 50 μm. Cresyl violet (CV) was used as counterstain in (**a**, **b**). Hematoxylin was used as counterstain in (**d, e, g-i, k-m**)

Since the C6 antisense MAC-blocking treatment has a protective effect on chronic relapsing EAE, we studied the role of MAC on the pathology of the mouse spinal cord. To this end, formalin-fixed, paraffin embedded spinal cord tissue, collected post-relapse (day 44 p.i.) from no drug chronic relapsing EAE (*n* = 4), C6 antisense-treated chronic relapsing EAE (*n* = 6) or healthy control (*n* = 6) mice was analyzed. As expected, the no drug mice had severe lesions within the ventrolateral columns. Lesions were demyelinated as they showed loss of LFB (Fig. 4a, c) and PLP signal (Fig. 4d, f), and inflammatory as they showed abundant eosinophilic cell infiltrates, CD3+ lymphocytes within the meninges (Additional file 2: Figure S4), and strong reactivity for IBA-1, a marker of microglia/macrophages (Fig. 4g, j). Immunostaining for C9, a marker for MAC deposition, revealed abundant reactivity within the ventral horns (Fig. 4k, l, n), the areas composed of motor neurons, suggesting a link between MAC and motor disability. In contrast, none of the C6 antisense-treated mice had demyelinated lesions (Fig. 4b, e). Quantification of LFB and PLP signal showed a trend towards decrease in the C6 antisense-treated mice compared to controls which is likely a result of acute disease (Fig. 4c, f). All the C6 antisense-treated mice showed IBA-1+ microglia of a resting morphology (Fig. 4h, i), amounts of IBA-1 reactivity similar to those found in controls (Fig. 4j) and lack of CD3+ lymphocytes in the parenchyma or the meninges (Additional file 2: Figure S4). Reactivity for C9 was sparse (Fig. 4m), consistent with a drastic reduction of MAC formation by the C6 antisense (~ 70%, ***p < 0.001; Fig. 4n).

**Fig. 5:**
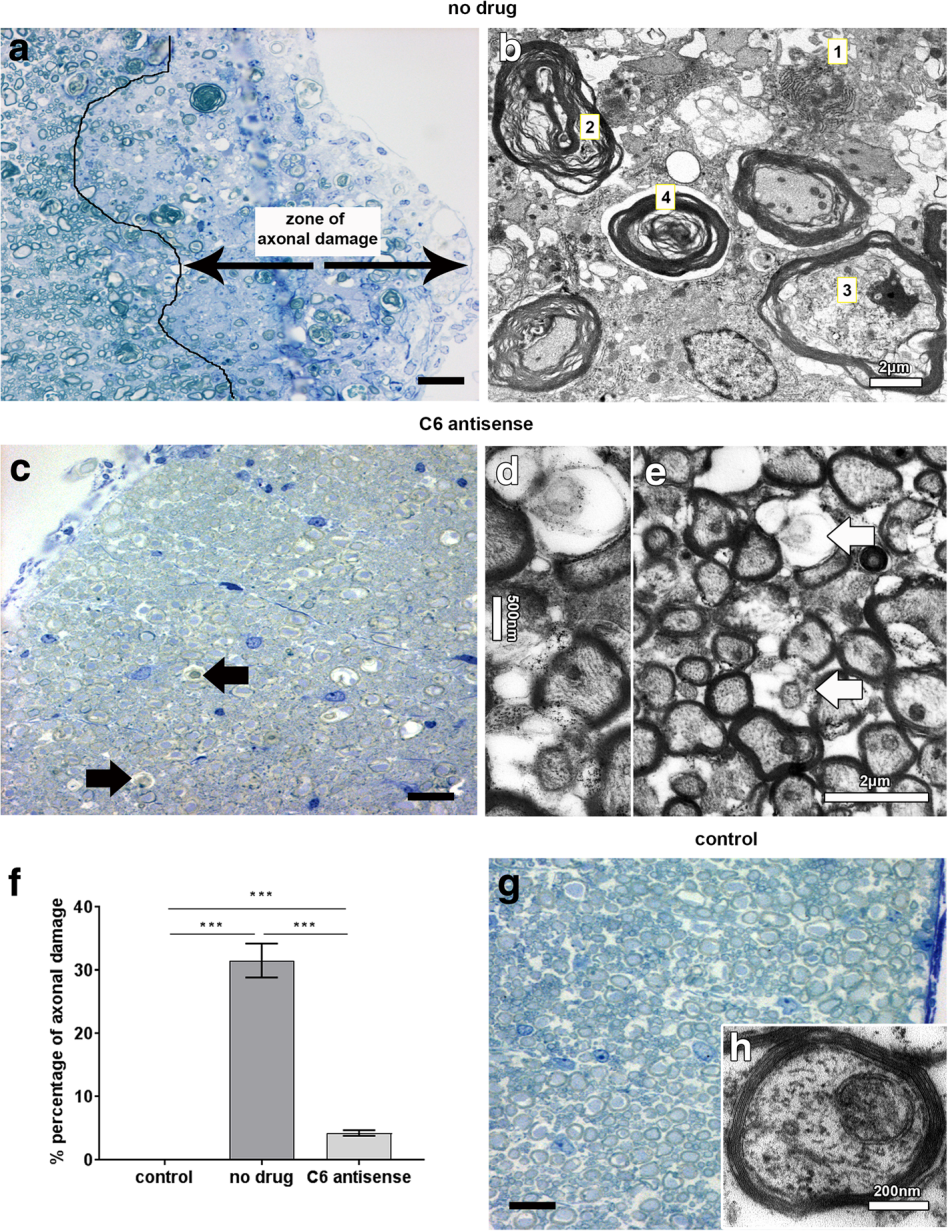
C6 antisense-mediated MAC inhibition protects from axonal damage. Richardson’s staining on spinal cord tissue from no drug mice showed wide areas of axonal damage corresponding to the lesioned areas (**a**). Electron microscopy micrograph indicates the types of axonal damage detected and quantified: 1. destruction of the myelin sheathe, 2. deformation of the myelin sheathe with gaps between tangent layers, 3. infoldings of the myelin sheathe within the axoplasmic area, and 4. onion bulbs (**b**). Picture of Richardson’s stained spinal cord section and electron microscopy micrograph from C6 antisense-treated mice (**c**-**e**) showing rarely detected damaged axons (arrows in **c, d** and arrows in **e**). Graph shows the percentage of axonal damage as quantified in the ventral and lateral column of the cervical and thoracic spinal cord segments from control (*n* = 6), no drug (*n* = 4) or C6 antisense-treated (*n* = 6) mice, collected post-relapse. Comparisons between groups were performed using the Kruskal-Wallis test by ranks. Data are expressed as the average (mean) ± SEM. Statistical differences are indicated (***p < 0.001) (**f**). Pictures from control mice showed intact axons only (**g**, **h**). Scale bars: (**a**, **c**, **g**) 25 μm, (**b**, **e**) 2 μm, (**d**) 500 nm (**h)** 200 nm

Next, we quantified axonal damage on osmium-fixed, epon-embedded spinal cord tissue, collected post-relapse (day 44 p.i.) from no drug chronic relapsing EAE (*n* = 4), C6 antisense-treated chronic relapsing EAE (*n* = 6) or healthy control (*n* = 6) mice. We report that all the no drug mice showed wide zones of axonal damage corresponding to the lesioned areas (Fig. 5a). Electron microscopy demonstrated various types of axonal damage that we detected and quantified; 1: destruction of the myelin sheath, 2: deformation of the myelin sheath with gaps between tangent layers, 3: infoldings of the myelin sheath within the axoplasmic area, and 4: onion bulbs (Fig. 5b). In contrast, the C6 antisense-treated mice examined had only a few damaged axons (arrows in Fig. 5c, d and arrows in Fig. 5e) and an average percentage of axonal damage that was significantly smaller than that found in the no drug littermates (C6 antisense vs no drug, 4.23 ± 0.44 vs 31.56 ± 2.67, mean ± SEM, ***p < 0.001; Fig. 5f). Control mice contained intact axons only (Fig. 5g, h).

**Fig. 6:**
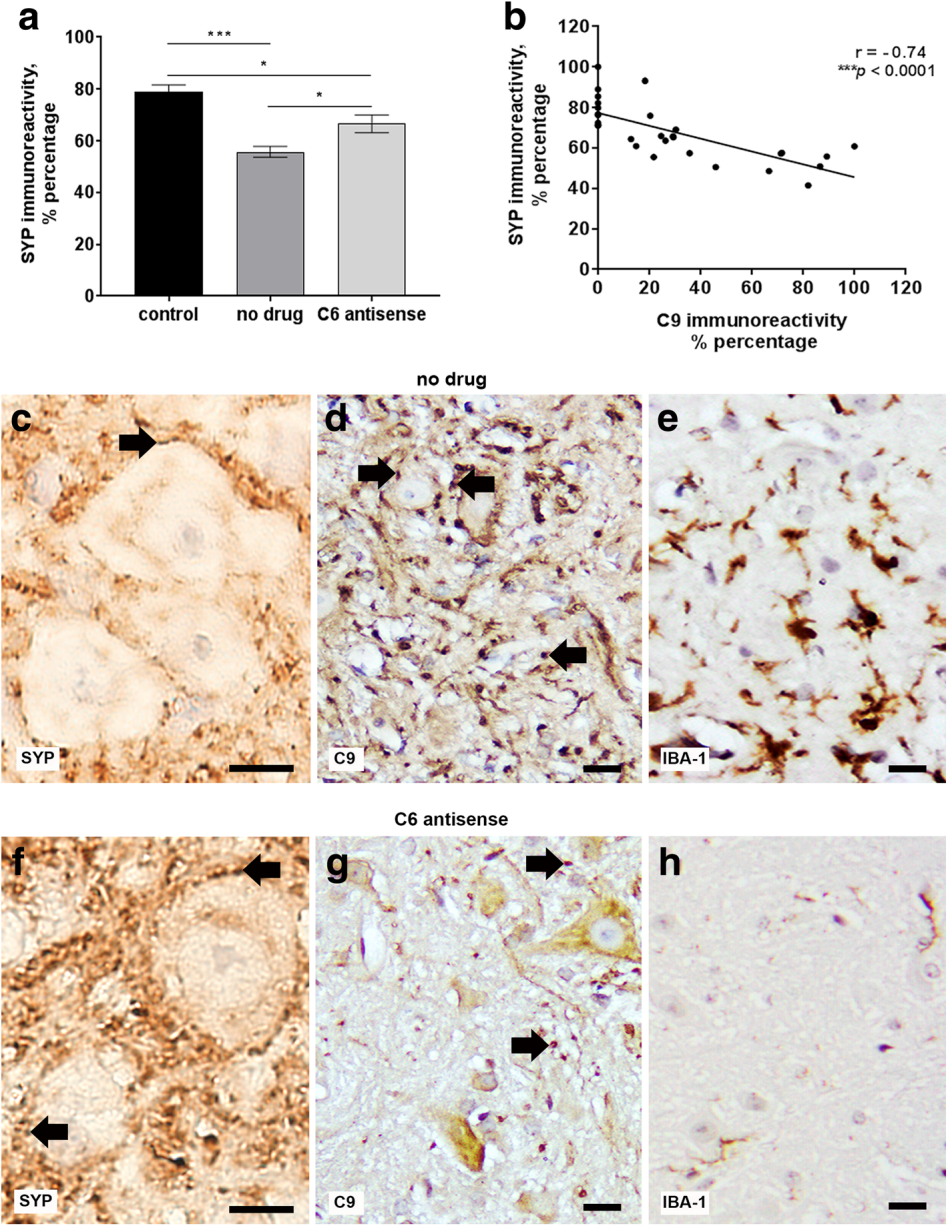
C6 antisense-mediated MAC inhibition protects from synaptic alterations. Quantification of synaptophysin (SYP) positive punctae showed a significant decrease of synaptic densities (*p < 0.05) in the no drug (*n* = 4) compared to the C6 antisense-treated mice (*n* = 6), post-relapse phase. Differences between groups were analyzed by using the One-Way Analysis of Variance test. Data are expressed as the average (mean) ± SEM. Statistical differences are indicated (*p < 0.05, ***p < 0.001). Controls are healthy mice injected with adjuvant only (*n* = 6) (**a**). Pearson’s correlation coefficient showed a significant negative correlation between SYP and the C9 marker of MAC (coefficient, *r* = – 0.74, ***p < 0.0001), in the mouse spinal cord (*n* = 29 corresponding fields plotted) (**b**). Histological analysis of paraffin spinal cord sections from no drug mice showed low densities of SYP+ punctae (arrow pointing to a synapse), sign of synaptic alterations or loss (**c**), abundant C9 reactivity (arrows) (**d**), and ionized calcium-binding adapter molecule 1 (IBA-1) positive microglia/macrophages with a morphology consistent with an activated status (**e**). In contrast, histological analysis of spinal cords from C6 antisense-treated mice showed abundant SYP+ punctae (arrows) (**f**), sparse C9 reactivity (arrows) (**g**), and IBA-1+ microglia with a morphology consistent with a resting status (**h**). Scale bars: (**c**-**h**) 10 μm. Hematoxylin was used as counterstain in (**c**-**h**)

Finally, we quantified the number of synapses and neurons in the grey matter of stereotactic sections of formalin-fixed spinal cords collected post-relapse (day 44 p.i.) from no drug chronic relapsing EAE (*n* = 4), C6 antisense-treated chronic relapsing EAE (*n* = 6) or healthy control (*n* = 6) mice. All the C6 antisense-treated mice showed higher levels of reactivity for SYP, a marker of synapses, compared with the no drug mice (C6 antisense vs no drug, 66.59 ± 3.49 vs 55.75 ± 2.06, mean ± SEM, *p < 0.05). Comparison with the controls indicated that the no drug mice had an average 23% decrease of SYP+ punctae (no drug vs control, 55.75 ± 2.06 vs 79.00 ± 2.60, mean ± SEM, ***p < 0.001), while in contrast, the C6 antisense-treated mice had a smaller 12% decrease (C6 antisense vs control, 66.59 ± 3.49 vs 79.00 ± 2.60, mean ± SEM, *p < 0.05) (Fig. 6a). Neuron counts showed no significant differences between the groups (*data not shown*), suggesting that any loss of SYP reactivity was not related to neuronal loss. Correlation analysis indicated a significant negative correlation between C9 and SYP (coefficient, *r* = − 0.74, ***p < 0.0001) (Fig. 6b), supporting a link between MAC deposition and synaptic changes. Histological examination of the ventral horns of no drug mice showed a punctate staining pattern for C9, indicative of a synaptic localization (Fig. 6d) that was further confirmed by the co-localization of the C9 and SYP markers (Additional file 2: Figure S5). Notably, deposition of C9 within the ventral horns of no drug mice was associated with the presence of abundant microglia or macrophages (Fig. 6e) and NLRP3 immunoreactivity (Additional file 2: Figure S6). Examination of the ventral horns of C6 antisense-treated mice showed abundant SYP+ punctae, few microglia of a resting phenotype and absence of NLRP3 reactivity (Fig. 6f-h and Additional file 2: Figure S6).

## Discussion

This study demonstrates that systemic knockdown of complement *C6* mRNA, when applied after the disease induction, prevents relapse in chronic relapsing EAE. Inhibition of MAC formation is the most likely mechanism of action of the C6 antisense oligonucleotide. Reduced amounts of MAC in the CNS are associated with lack of activation of local parenchymal inflammatory pathways known to induce or maintain neuroinflammation, such as the NLRP3 inflammasome. Protection against clinical disability conferred by MAC inhibition is exerted through the prevention of axonal and synaptic damage. Overall, these data suggest that even in the presence of activated T lymphocytes, MAC is a key contributor to neuroinflammation driving degeneration. Inhibition of C5aR1-mediated inflammation also affected disease severity but only ameliorated progressive neurological disability.

The complement system, a major component of innate immunity, is a sensor of immune homeostasis and a versatile effector of host defense. The classical pathway of complement activation involves recognition of pathogen- or danger-associated molecular patterns by C1q, induction of pro-inflammatory responses by cleaved C3 and C5, and elimination of targeted cells by MAC. While complement proteins are rapidly recruited to protect the human body against intruders, this pathway may also transform to an unwanted self-attacking mechanism that perpetuates injury if uncontrolled [66].

Because of the central role of complement in immunity, the interest in anti-complement therapeutics has increased over the last decades. Multiple agents targeting different components of the complement system have been developed [57]. The terminal pathway of complement is undoubtedly an interesting signaling branch for intervention. Inhibition at this level prevents potentially deleterious inflammatory responses without affecting upstream complement functions that are beneficial [75]. Eculizumab (Soliris; Alexion Pharmaceuticals) is an inhibitor of the C5 component and the only terminal pathway-specific drug that has so far received approval for clinical use. Soliris is prescribed in patients with paroxysmal nocturnal hemoglobinuria (PNH) or atypical hemolytic uremic syndrome (aHUS) [27, 84] and is currently undergoing phase III of a clinical trial for the prevention of neuromyelitis optica (NMO) relapses (https://clinicaltrials.gov/ct2/show/NCT01892345). It targets C5, blocking both MAC assembly and the formation of C5a [27, 84].

MAC is the end product of complement activation and the most inflammatory component of the terminal complement pathway [66]. Lytic levels of MAC can directly destroy infected or damaged cells, by disrupting plasma membranes, which are then cleared by recruited phagocytes [20, 30]. Moreover, sub-lytic amounts of MAC can trigger the activation of NLRP3 inflammasome in the target cells, promoting clinical disease and primary or secondary tissue damage [45, 78].

On the other hand, C5a is known to have both pro-and anti-inflammatory properties depending on the receptor-ligand interaction and disease studied [9, 47]. More specifically, C5a can bind to one of the two competing receptors, C5aR1 (CD88) and C5aR2 (C5L2), and the balance in C5a occupancy determines whether the response is pro- or anti-inflammatory [81, 87]. Pro-inflammatory responses include G-protein-dependent cell signaling leading to intracellular Ca^+ 2^ production, chemotaxis and oxidative stress. Anti-inflammatory responses include non G-protein-dependent signaling that alters pro-inflammatory cytokine production, limiting inflammation [46, 81].

Thus far, there is no complete understanding of the role of the terminal complement pathway on neuroinflammation. A number of studies showing deletion or pharmacological inhibition of complement components, related fragments or complement receptors have been published reporting controversies. More specifically, deficiency or blockade of C5a receptors failed to protect against acute EAE, while expression of C5a in the brain did not exacerbate EAE [56, 64, 65]. Moreover, deficiency of C5 reduced remyelination in chronic EAE possibly due to an effect of MAC on oligodendrocyte apoptosis [58, 83]. In contrast, deficiency of C6 in a rat model of acute EAE protected against demyelination and immune cell infiltration [51, 77]. Notably, reconstitution of C6 in these rats restored EAE [51].

In this study, we aimed to define the role of MAC on neuroinflammation by delineating its effect from that induced by C5aR1, and compare the effect of a MAC inhibitor, being a knockdown of C6, to that of a C5aR1 antagonist. We found that systemic administration of the MAC inhibitor after disease onset prevented relapse in mice with chronic relapsing EAE by suppressing key pro-inflammatory pathways within the CNS. In contrast, administration of the C5aR1 antagonist only mitigated neurological disability.

Which factor determines the molecular and/or clinical differences between the two treatment groups? Expression profiling of the spinal cords of the chronic relapsing EAE animals shows that C6 inhibition has a major effect on the NLRP3 inflammasome pathway. The NLRP3 inflammasome is a multiprotein complex abundantly expressed in the CNS that is activated by a wide range of endogenous or exogenous signals to potentiate neuroinflammatory responses with the release of IL-1β and IL-18 [79]. Activation of the inflammasome was previously linked to MAC deposition [45, 78]. MAC precursor proteins can leak into the CNS during transient openings of the blood–spinal cord barrier (BSCB) caused by immunization with spinal cord antigens in the chronic relapsing EAE model [71]. Our RNA-seq data demonstrated that the NLRP3 inflammasome pathways are ‘switched on’ in the no drug and the C5aR1-inhibited mice, but not in the MAC-inhibited mice in comparison with controls. Moreover, pathway analysis with IPA showed that the Tec kinase signaling, a regulator of the NLRP3 inflammasome [34], was upregulated in the C5aR1-inhibited mice, but not in the MAC-inhibited littermates. These data were consistent with our finding that all the no drug and the C5aR1-inhibited mice showed IL-1β/pro-IL-1β + glial cells while, none of the C6 antisense-treated mice showed any IL-1β/pro-IL-1β immunostaining. Our data confirm previous reports of a specific effect of MAC on the activation of NLRP3 inflammasome [45, 78] and further suggest that MAC formation stimulates immune responses in glial cells to initiate or boost neuroinflammation in this model.

Indeed, examination of spinal cords from no drug mice showed MAC deposition associated with abundant microglia/macrophages, sign of local immune reaction to MAC. MAC was deposited on synapses and its reactivity was negatively correlated to synaptic densities. In contrast, spinal cords of MAC-inhibited mice showed a few microglia of a resting phenotype and higher synaptic densities. These data together with our observation that MAC deposits are restricted to the ventral horns, the area of motor neurons, and are linked to local NLRP3 expression suggest a link between MAC, accumulation and/or activation of microglia and neurological disability in chronic relapsing EAE. It is possible that deposition of MAC on synapses leads to synaptic damage due to pore formation. Pores may in turn, promote the release of ATP [59], an important messenger that can be sensed by the neighboring glia via the purinoceptor 7 (P2X7) receptor [49], to induce bystander NLRP3 inflammasome activation for release of IL-1β. IL-1β can then exacerbate disease by triggering the opening of the BSCB and promoting the infiltration of immune cells in the CNS [73].

Once peripheral immune cells enter the CNS parenchyma, complement can regulate their differentiation, in the case of lymphocytes [2, 12], or promote their activation by the release of anaphylatoxins, in the case of macrophages or neutrophils [25]. Activated immune cells are important mediators of neuroinflammation and lesion formation. Importantly, we found only a few immune cells, no demyelinated lesions and a small number of damaged axons in all the MAC-inhibited mice tested while, in contrast, all the no drug mice showed demyelinated lesions with abundant cell infiltrates and damaged axons. These data are in line with our previous finding that MAC inhibition reduces the numbers of infiltrating macrophages, impeding degeneration in the CNS after brain trauma [17, 69]. In addition, C6 deficient rats (rats genetically unable to form MAC) showed reduced numbers of CD68+ and CD11b + cells at the site of injury after crush nerve injury [63].

Comparison of RNA-seq data from MAC-inhibited mice to those from no drug mice showed activation of two important anti-inflammatory pathways, the PPAR and the LXR/RXR pathway [15, 37, 76]. It is known that the C6 antisense oligonucleotide does not penetrate the CNS. Therefore, we speculate that activation of these protective mechanisms in the MAC-inhibited mice might be an intra-parenchymal response that is related either to blockade of immune cell infiltration or to another unknown function of the C6 antisense oligonucleotide. In contrast, the C5aR1-inhibited mice showed activation of the PPAR pathway only. This is an interesting observation since the C5aR1 antagonist enters the CNS and may therefore act both in the periphery and the CNS.

MAC has been involved in the pathology of a wide range of acute and chronic neuroinflammatory diseases. Acute multiple sclerosis, stroke, traumatic brain injury (TBI) and NMO are major examples of acute or recurrent neuroinflammatory brain conditions in which MAC may have an important pathological contribution [6, 17, 48, 54]. Moreover, MAC, detected by an antibody against a neo-epitope in C9 (C9neo), was found in brains from donors with the chronic course of multiple sclerosis, in the absence of fulminate inflammation [82], in Alzheimer’s disease brains on senile plaques [50, 55], and in amyotrophic lateral sclerosis (ALS) brains on still innervated motor endplates, suggesting a role as a modifier of disease progression [3]. Although chronic diseases are far less inflammatory than acute, their pathology does include inflammatory responses, but of a type different than the acute ones, and this might explain the failure of current treatments to halt disease progression [42]. Our finding in mice that MAC is linked to NLRP3 inflammasome activation and IL-1β production might have implications for the diagnosis and therapy of neuroinflammation-related diseases in humans, especially in the view of accumulating evidence pointing towards a pathogenic role of IL-1β. IL-1β transcript or protein was detected in the cerebrospinal fluid, blood or lesions of patients with multiple sclerosis [11, 26, 60] and was linked to the extent of cortical demyelination or to disease severity [68, 72]. Interestingly, treatment of multiple sclerosis patients with the FDA-approved therapeutic agents type-I IFN, glatiramer acetate or natalizumab (https://www.ncbi.nlm.nih.gov/books/NBK294198/table/introduction.t1/#__NBK294198_dtls__) was shown to affect the expression of IL-1Ra and/or the production of IL-1β [10, 23, 52, 53].

Complement inhibition, as with most immunomodulatory interventions, weakens the immune responses to pathogens, which over time, can lead to recurrent and/or severe infections in patients. Although activation of upstream complement functions suffices for protection against most infective organisms, there is one group of bacteria, the *Neisseria* species, which requires activation of the terminal pathway [16]. *Neisseria meningitides* infection leads to meningococcal sepsis or meningitis. Individuals deficient in C5 or terminal pathway components have a higher susceptibility for *Neisseria meningitides* infections. However, not all deficiencies might have similar increased risks. C6 and C7 deficiencies could be less severe [86]. For prevention of meningococcal disease it is recommended that patients receiving Soliris receive prophylactic antibiotics and are vaccinated against all meningococcal strains [18]. It remains to be demonstrated whether treatment with a C6 inhibitor will cause less risk of meningococcal infections due to the extra anti-microbial protection provided by the C5a-related anaphylactic mechanism. Therapeutic anti-C6 antibodies are now under development and in a preclinical stage [57]. These antibodies may serve to circumvent problems related to blockade of C5a-mediated inflammation.

In this study, we also show that PMX205, a C5aR1 inhibitor ameliorates disability but does not completely prevent the relapse in chronic relapsing EAE. An explanation for this difference is that MAC is a more potent pro-inflammatory molecule in this model than C5a. In addition, C5a does not have only pro-inflammatory but it also has anti-inflammatory properties [9]. Compared to the no drug mice, there is a clear effect of PMX205 however. The severity of the relapse was less. Expression profiling showed reduced expression of inflammatory pathways regulating relapse in chronic relapsing EAE. However, activation of the NLRP3 inflammasome, as measured by expression levels of related genes and by IL-1β/pro-IL-1β + glial cells, was moderately affected. It is therefore likely that MAC predominantly drives this effect in this model.

A limitation of this study is the lack of data related to the contribution of the two tested inhibitors on the mouse peripheral cell immunity. Further research is required to address impact on immune cells peripherally and in the CNS. Our findings add to previous reports of a link between the terminal complement pathway and inflammasome activation. In this study we further demonstrate that MAC is a major player in neuroinflammation that drives spinal cord degeneration, providing a rationale for the use of complement-targeting therapies to combat chronic neuroinflammatory diseases.

## Conclusions

Our research demonstrates that inhibition of the terminal complement pathway at the symptomatic disease phase effectively stops neuroinflammation in the chronic relapsing EAE model. This is the first study that delineates the specific roles of C5a receptor signaling and MAC formation in vivo using selective inhibitors. Our finding that systemic inhibition of MAC prevented relapse completely in mice whereas, inhibition of the C5aR1 mitigated neurological disability reveals a key role for MAC in neuroinflammation driving degeneration. This study might represent a first step towards pre-clinical development of MAC inhibition as therapy for chronic neuroinflammatory diseases like multiple sclerosis.

## Additional files

Additional file 1: **Table S1.** Expression levels of key immune genes in mice with chronic relapsing EAE showing mild or severe neurological disability and comparison with mice showing no disability. **Table S2.** Mouse primer sequences. **Table S3.** Primary antibodies, dilution, source. **Table S4.** Log fold change values of NLRP3 inflammasome genes in the mouse spinal cord at the relapse of chronic relapsing EAE. (PDF 215 kb)

Additional file 2: **Figure S1.** Systemic administration of C6 antisense knocks down C6 in mice. **Figure S2.** QPCR determination of key components of the inflammasome pathway. **Figure S3.** PMX205 concentrations in plasma, brain and spinal cord. **Figure S4.** Systemic administration of C6 antisense prevents inflammation in the spinal cord of the chronic relapsing EAE model. **Figure S5.** C9 is localized at synapses. **Figure S6.** Systemic administration of C6 antisense prevents NLRP3 inflammasome expression in the spinal cord of the chronic relapsing EAE model. (PDF 877 kb)
