# Supplementary material for: Systemic inhibition of the membrane attack complex impedes neuroinflammation in chronic relapsing experimental autoimmune encephalomyelitis
Source: Acta Neuropathol Commun. 2018 May 3;6:36. doi: 10.1186/s40478-018-0536-y (PMC5932802; doi:10.1186/s40478-018-0536-y)
Supplement: Supplementary file 1 — Table S1. Expression levels of key immune genes in mice with chronic relapsing EAE showing mild or severe neurological disability and comparison with mice showing no disability. Table S2. Mouse primer sequences. Table S3. Primary antibodies, dilution, source. Table S4. Log fold change values of NLRP3 inflammasome genes in the mouse spinal cord at the relapse of chronic relapsing EAE. (PDF 215 kb) [file 40478_2018_536_MOESM1_ESM.pdf]

Systemic inhibition of the membrane attack complex impedes neuroinflammation in chronic relapsing experimental autoimmune encephalomyelitis

Iliana Michailidou[1,2], Aldo Jongejan[3] , Jeroen P. Vreijling[2], Theodosia Georgakopoulou[1], Marit B. de Wissel[1], Ruud A. Wolterman[1], Patrick Ruizendaal[1], Ngaisah Klar-Mohamad[4], Anita E. Grootemaat[5], Daisy I. Picavet[5], Vinod Kumar[6], Cees van Kooten[4], Trent M. Woodruff[6], B. Paul Morgan[7], Nicole N. van der Wel[5], Valeria Ramaglia[1,8], Kees Fluiter[2†] and Frank Baas[2†*]

[1]Department of Genome Analysis, Academic Medical Center, Amsterdam, The Netherlands; [2]Department of Clinical Genetics, Leiden University Medical Center, Leiden, The Netherlands, [3]Department of Bioinformatics, Academic Medical Center, Amsterdam, The Netherlands; [4]Department of Nephrology, Leiden University Medical Center, Leiden, The Netherlands; [5]Electron Microscopy Centre Amsterdam, Department of Medical Biology, Academic Medical Center, Amsterdam, The Netherlands; [6]School of Biomedical Sciences, The University of Queensland, Brisbane, Australia; [7]Systems Immunity University Research Institute, School of Medicine, Cardiff University, Cardiff, UK; [8]Department of Immunology, University of Toronto, Toronto, Canada.

[†] authors contributed equally to this study.

*Corresponding author: Prof Dr Frank Baas, Department of Clinical Genetics, Leiden University Medical Center, Einthovenweg 20, 2333 ZC Leiden, The Netherlands. Tel: 0031 71 526 9868. E-mail: F.Baas@lumc.nl

Table S1 Expression levels of key immune genes in mice with chronic relapsing EAE showing mild or severe neurological disability and comparison with mice showing no disability

GenBank ID	Symbol	Description / Full name	Av. Express. No Disability	Av. Express. Mild Disability	Av. Express. Severe Disability
Complement System					
NM_009778	C3	complement component 3	0.113	0.152, *p*=ns	**0.843, ******p*<0.001**
NM_016704	C6	complement component 6	0.154	0.256, *p*=ns	**1.247, ******p*<0.001**
NM_010016	Cd55	decay accelerating factor for complement	1.549	1.523, *p*=ns	1.465, *p*=ns
NM_009776	Serping1 (C1INH)	serine (or cysteine) peptidase inhibitor, clade G, member 1	0.163	0.201, *p*=ns	**1.104, ******p*<0.001**
Inflammasomes					
NM_001004142	Nlrp1a	NLR family, pyrin domain containing 1A	0.079	0.078, *p*=ns	**0.965, ******p*<0.001**
NM_145827	Nlrp3	NLR family, pyrin domain containing 3	0.055	0.071, *p*=ns	**0.834, ******p*<0.001**
NM_001013779	Aim2	absent in melanoma 2	0.513	1.406, ****p*=0.05**	0.825, *p*=ns
NM_023258	Pycard (Asc)	PYD and CARD domain containing	0.241	0.236, *p*=ns	**0.885, ******p*<0.001**
NM_001033367	Nlrc4	NLR family, CARD domain containing 4	0.049	0.050, *p*=ns	**0.872, ******p*<0.001**
NM_008361	Il1β	interleukin 1 beta	0.006	0.007, *p*=ns	**0.601, ******p*<0.001**
Monocyte/Macrophage activation					
NM_031195	Msr1	macrophage scavenger receptor 1	0.006	0.007, *p*=ns	**0.527, ******p*<0.001**
NM_009841	Cd14	antigen expressed in	0.025	0.029, *p*=ns	**0.681, ******p*<0.001**

		monocyte/macrophages			
NM_007643	Cd36	antigen expressed in monocyte/macrophages	0.026	0.018, *p*=ns	**0.852, **p<0.01**
NM_011905	Tlr2	toll-like receptor 2	0.037	0.044, *p*=ns	**0.790, ***p<0.001**
NM_021297	Tlr4	toll-like receptor 4	0.051	0.062, *p*=ns	**0.754, ***p<0.001**
NM_031168	Il6	interleukin 6	0.012	0.012, *p*=ns	**0.644, ***p<0.001**
NM_008689	NFkβ1	nuclear transcription factor	0.223	0.238, *p*=ns	**0.856, ***p<0.001**
NM_011333	Ccl2	Ccl2 chemokine (C-C motif) ligand 2	0.005	0.005, *p*=ns	**0.728, **p<0.01**
Astroglia activation					
NM_001131020	Gfap1	glial fibrillary acidic protein	0.360	0.419, *p*=ns	**0.849, ***p<0.001**
LXR/RXR pathway					
NM_013839	Nr1h3 (lxra)	nuclear receptor subfamily 1, group H, member 3	0.297	0.330, *p*=ns	**0.892, ***p<0.001**
NM_009473	Nr1h2 (lxrb)	nuclear receptor subfamily 1, group H, member 2	0.766	0.843, *p*=ns	**1.022, ***p<0.001**
NM_013454	Abca1	ATP-binding cassette, sub-family A (ABC1), member 1	0.096	0.098, *p*=ns	**0.582, ***p<0.001**
NM_009696	Apoe	apolipoprotein E	0.207	0.268, *p*=ns	**0.821, ***p<0.001**
NM_011305	Rxra	retinoid X receptor alpha	0.513	0.604, *p*=ns	**0.781, ***p<0.001**
NM_009107	Rxrg	retinoid X receptor gamma	1.336	1.458, *p*=ns	1.237, *p*=ns

No Disability: clinical score=0; Mild Disability: 0<clinical score≤3; Severe Disability: 3<clinical score≤5. Statistically significant values are marked in bold. *ns*: non-significant

Table S2 Mouse primer sequences

Target gene	Primer	Sequence
C3	Forward	accttacctcggcaagtttct
	Reverse	ttgtagagctgctggtcagg
C6	Forward	cagagaaaaatgaacattcccatta
	Reverse	ttcttgtgggaagctttaatgac
Cd55	Forward	actgttgattgggacgatgag
	Reverse	tggtggctctggacaatgta
Serping1 (C1INH)	Forward	ccaaaggtgtcacttctgtgtc
	Reverse	gagatgcattcacataggtgtcc
Nlrp1a	Forward	agacgttgtcccccatgtt
	Reverse	ggtgggtcaggctgtctcta
Nlrp3	Forward	gaattccggccttacttcaa
	Reverse	ggtgtgtgaagttctggttgg
Aim2	Forward	tgggctgtttaaagtccagaa
	Reverse	cacctccattgtccctgttt
Pycard (Asc)	Forward	ccagagttctgtttcttaccttgag
	Reverse	tcacagcccagagttagtatgc
Nlrc4	Forward	ctgcaagaacatgcataccc
	Reverse	tactgttcgtcatccgtggt
Il1β	Forward	agttgacggaccccaaaag
	Reverse	agctggatgctctcatcagg
Msr1	Forward	ctggacaaactggtccacct
	Reverse	gtccccgatcacctttaaca
Cd14	Forward	aaagaaactgaagcctttctcg
	Reverse	agcaacaagccaagcacac
Cd36	Forward	ttgaaaagtctcggacattgag
	Reverse	tcagatccgaacacagcgta
Tlr2	Forward	ggggcttcacttctctgctt
	Reverse	agcatcctctgagatttgacg
Tlr4	Forward	ggactctgatcatggcactg
	Reverse	ctgatccatgcattggtaggt

Il6	Forward	gctaccaaactggatataatcagga
	Reverse	ccaggtagctatggtactccagaa
NFkβ1	Forward	ctgctcaggtccactgtctg
	Reverse	tgtcactatcccggagttca
Ccl2	Forward	catccacgtgttggctca
	Reverse	gatcatcttgctggtgaatgagt
Gfap1	Forward	cagcttacggccaacagtg
	Reverse	gcctcaggttggtttcatct
Nr1h3 (lxra)	Forward	gagtgtcgacttcgcaaatg
	Reverse	cggatctgttcttctgacagc
Nr1h2 (lxrb)	Forward	tgccagggttcttgcagt
	Reverse	agcgtctggctgtttctagc
Abca1	Forward	ttccatcctccttgtcatctc
	Reverse	tccttggcaaagttcacaaa
Apoe	Forward	ttggtcacattgctgacagg
	Reverse	agcgcaggtaatcccagaa
Rxra	Forward	acatgcagatggacaagacg
	Reverse	gggtttgagagccccttaga
Rxrg	Forward	cagaagtgcctggtcatgg
	Reverse	cctcactctctgctcgctct
HPRT	Forward	ggtccattcctatgactgtagatttt
	Reverse	caatcaagacgttctttccagtt

Table S3 Primary antibodies, dilution, source

**Antigen**	**Clone**	**Dilution/Concentration**	**Source**
PLP	plpc1	0.3μg/ml [a]	Serotec
MAG	Monoclonal (#ab89780)	1:1,000 [c]	Abcam
IBA-1	Polyclonal	1:3,000 [c]	Wako
CD3	SP7	1:500 [b]	Abcam
C9	Polyclonal	1:200 [c]	Made in house (BPM, Cardiff)
SYP	YE269	1:600 [b]	Abcam
SYP	SVP-38	1:100 [b]	Sigma
IL-1β/pro-IL-1β	Polyclonal	1:70	Santa Cruz
NLRP3	Polyclonal	1:100 [b]	Abcam

Antigen retrieval of paraffin sections was performed by heat in [a] 0.05 M Tris buffered saline pH 7.6; [b] 10 mM citric acid buffer pH 6.0; [c] 10 mM Tris/1 mM EDTA buffer pH 9.0.

Table S4 Log fold change values of NLRP3 inflammasome genes in the mouse spinal cord at the relapse of chronic relapsing EAE

Gene	no drug *vs* control	C6 antisense *vs* control	PMX205 *vs* control
CASP1	15,319	0,666	12,685
IL-1β	19,370	-0,865	14,348
MYD88	18,952	0,873	0,731
NAIP	18,796	-0,162	0,906
NLRC4	12,786	0,576	0,698
NLRP1	15,981	-0,831	0,165
NLRP3	22,853	-0,484	15,752
PYCARD	1,349	-0,802	-0,181
TLR4	15,428	0,379	11,011

no drug: non-treated mice with chronic relapsing EAE; C6 antisense: mice with chronic relapsing EAE treated with the C6 antisense oligonucleotide; PMX205: mice with chronic relapsing EAE treated with the PMX205 antagonist of C5aR1; control: healthy naïve mice.
