# Supplementary material for: Systemic inhibition of the membrane attack complex impedes neuroinflammation in chronic relapsing experimental autoimmune encephalomyelitis
Source: Acta Neuropathol Commun. 2018 May 3;6:36. doi: 10.1186/s40478-018-0536-y (PMC5932802; doi:10.1186/s40478-018-0536-y)
Supplement: Supplementary file 2 — Figure S1. Systemic administration of C6 antisense knocks down C6 in mice. Figure S2. QPCR determination of key components of the inflammasome pathway. Figure S3. PMX205 concentrations in plasma, brain and spinal cord. Figure S4. Systemic administration of C6 antisense prevents inflammation in the spinal cord of the chronic relapsing EAE model. Figure S5. C9 is localized at synapses. Figure S6. Systemic administration of C6 antisense prevents NLRP3 inflammasome expression in the spinal cord of the chronic relapsing EAE model. (PDF 877 kb) [file 40478_2018_536_MOESM2_ESM.pdf]

Systemic inhibition of the membrane attack complex impedes neuroinflammation in chronic relapsing experimental autoimmune encephalomyelitis

Iliana Michailidou[1,2], Aldo Jongejan[3] , Jeroen P. Vreijling[2], Theodosia Georgakopoulou[1], Marit B. de Wissel[1], Ruud A. Wolterman[1], Patrick Ruizendaal[1], Ngaisah Klar-Mohamad[4], Anita E. Grootemaat[5], Daisy I. Picavet[5], Vinod Kumar[6], Cees van Kooten[4], Trent M. Woodruff[6], B. Paul Morgan[7], Nicole N. van der Wel[5], Valeria Ramaglia[1,8], Kees Fluiter[2†] and Frank Baas[2†*]

[1]Department of Genome Analysis, Academic Medical Center, Amsterdam, The Netherlands; [2]Department of Clinical Genetics, Leiden University Medical Center, Leiden, The Netherlands, [3]Department of Bioinformatics, Academic Medical Center, Amsterdam, The Netherlands; [4]Department of Nephrology, Leiden University Medical Center, Leiden, The Netherlands; [5]Electron Microscopy Centre Amsterdam, Department of Medical Biology, Academic Medical Center, Amsterdam, The Netherlands; [6]School of Biomedical Sciences, The University of Queensland, Brisbane, Australia; [7]Systems Immunity University Research Institute, School of Medicine, Cardiff University, Cardiff, UK; [8]Department of Immunology, University of Toronto, Toronto, Canada.

[†] authors contributed equally to this study.

*Corresponding author: Prof Dr Frank Baas, Department of Clinical Genetics, Leiden University Medical Center, Einthovenweg 20, 2333 ZC Leiden, The Netherlands. Tel: 0031 71 526 9868. E-mail: F.Baas@lumc.nl

Figure S1.

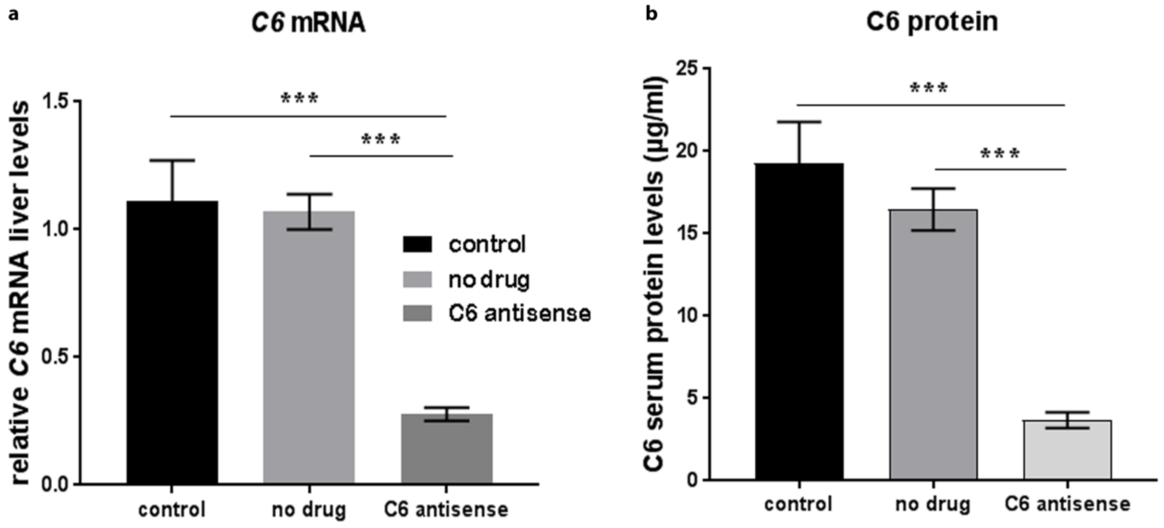

Fig. S1 Systemic administration of C6 antisense knocks down C6 in mice. Graph shows a 75% (***p<0.001) knockdown of *C6* mRNA in the C6 antisense-treated (n=13) compared with the no drug (n=17) mice as measured by qPCR in the liver (**a**). Graph shows a significant decrease (~80%, ***p<0.001) of C6 protein in the C6 antisense-treated (n=13) compared with the no drug (n=17) mice as measured by ELISA in the serum (**b**). Control mice (n=12) are adjuvant-only or naïve mice. Differences between groups were analyzed by using the One-Way Analysis of Variance test. Data are expressed as the average (mean)±SEM. Statistical differences are indicated (***p<0.001).

Figure S2.

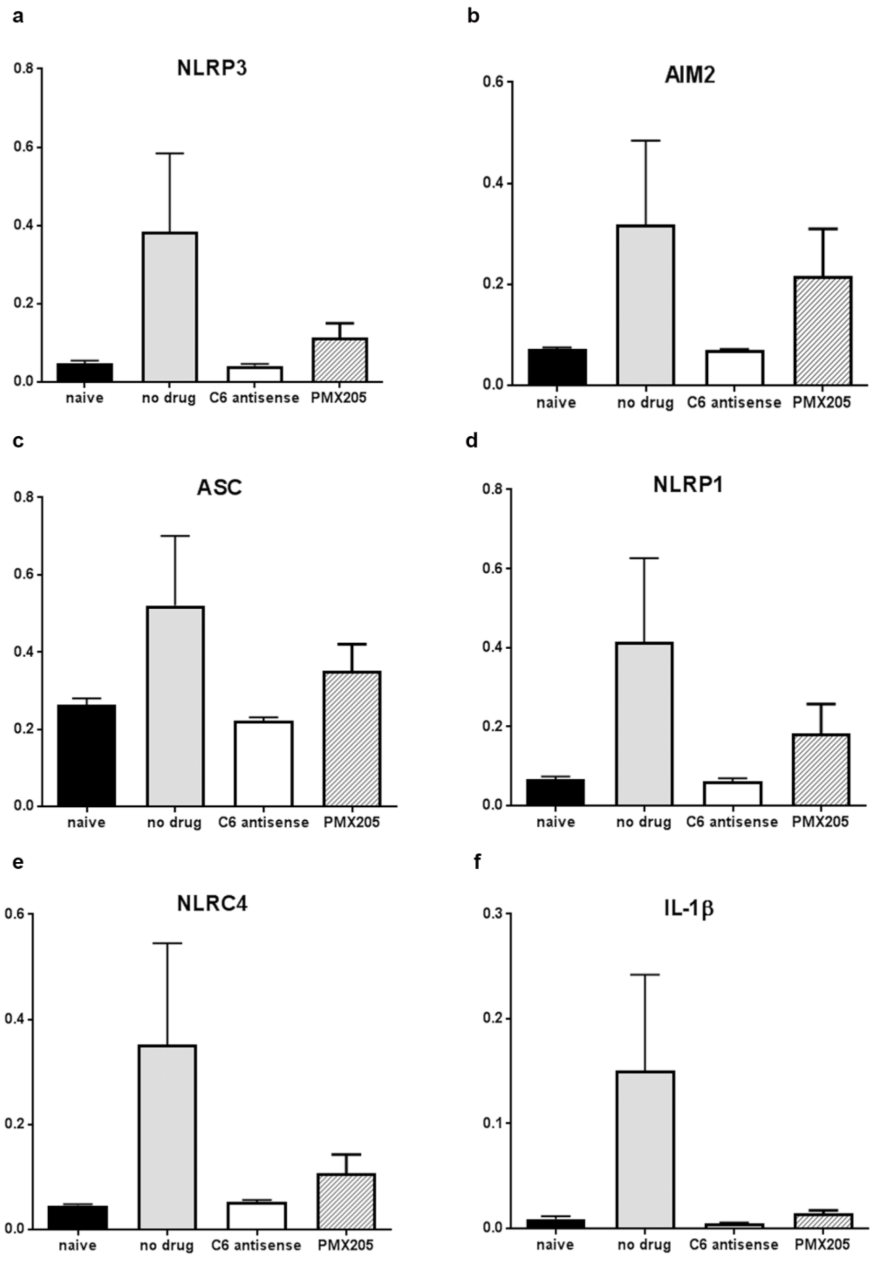

Fig. S2 QPCR determination of key components of the inflammasome pathway. Data were obtained from RNA-seq of mouse spinal cords collected at relapse (3 mice/group). Data are expressed as the average (mean)±SEM of three reactions after normalization with the housekeeping gene hypoxanthine phosphoribosyl transferase (HPRT). NLRP3, Nod-like receptor protein 3 (**a**); AIM2, Absent in melanoma 2 (**b**); ASC, apoptosis-associated speck-like protein containing a carboxy-terminal CARD (**c**); NLRP1, Nod-like receptor protein 1 (**d**); NLRC4, Nod-like receptor (NLR) family CARD domain containing protein 4 (**e**); IL-1β, interleukin 1 beta (**f**).

Figure S3.

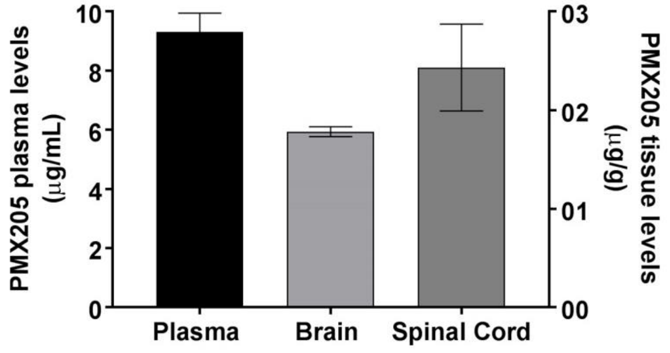

Fig. S3 PMX205 concentrations in plasma, brain and spinal cord. C57BL/6J mice (males; age 10-12 weeks; n=4) were administered 1 mg/kg of PMX205 by intraperitoneal injection. After 15 min, samples were collected, processed and analyzed using an LC-MS/MS method. Data are expressed as the average (mean)±SEM.

Figure S4.

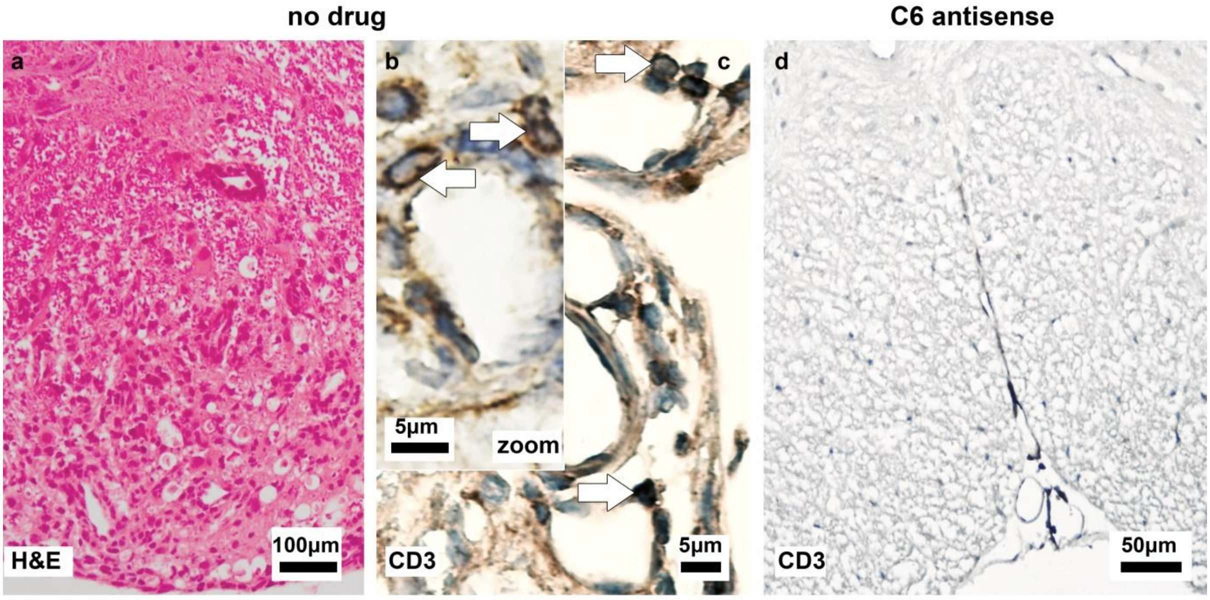

Fig. S4 Systemic administration of C6 antisense prevents inflammation in the spinal cord of the chronic relapsing EAE model. Hematoxylin and eosin (H&E) staining showed abundance of cells within the lesions of no drug mice (n=4) (**a**) while, in addition, immunostaining for the CD3 marker of lymphocytes, showed CD3+ cells in the meninges (**b**, **c**), both signs of inflammation. In contrast, all the C6 antisense-treated mice tested (n=6) showed lack of CD3+ lymphocytes (**d**). Tissue was collected post-relapse phase. Scale bars: (**a**) 100 μm, (**d**) 50 μm, (**b**, **c**) 5 μm. Hematoxylin was used as counterstain in (**a**-**d**).

Figure S5.

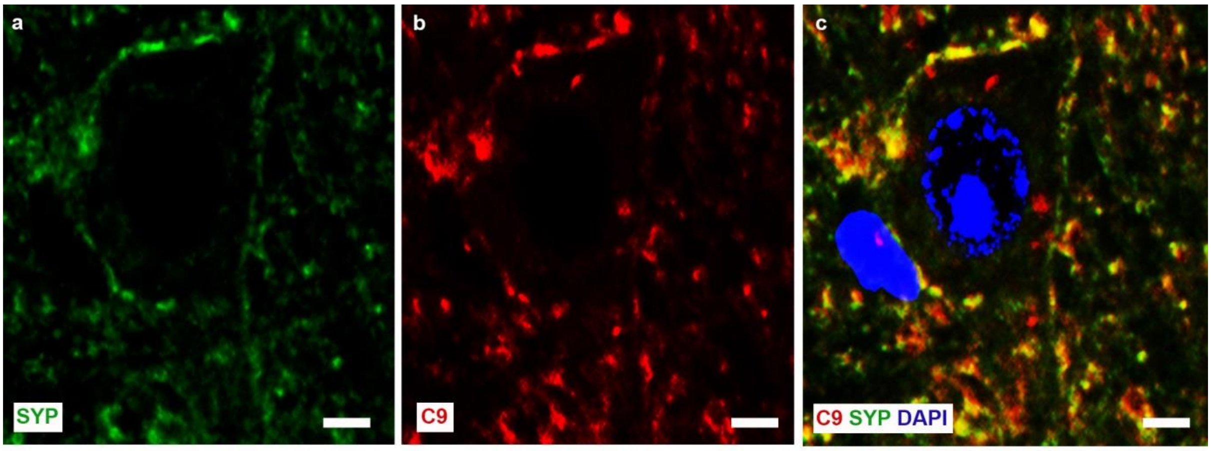

Fig. S5 C9 is localized at synapses. Double immunolabelling of synaptophysin (SYP, in green), marker of synapses (**a**), and C9 (in red), marker of the MAC (**b**), showed co-localization (**c**). Nuclei in (**c)** were stained with 4',6-diamidino-2-phenylindole (DAPI, blue). MAC, membrane attack complex. Scale bars: (**a**-**c**) 5 µm.

Figure S6.

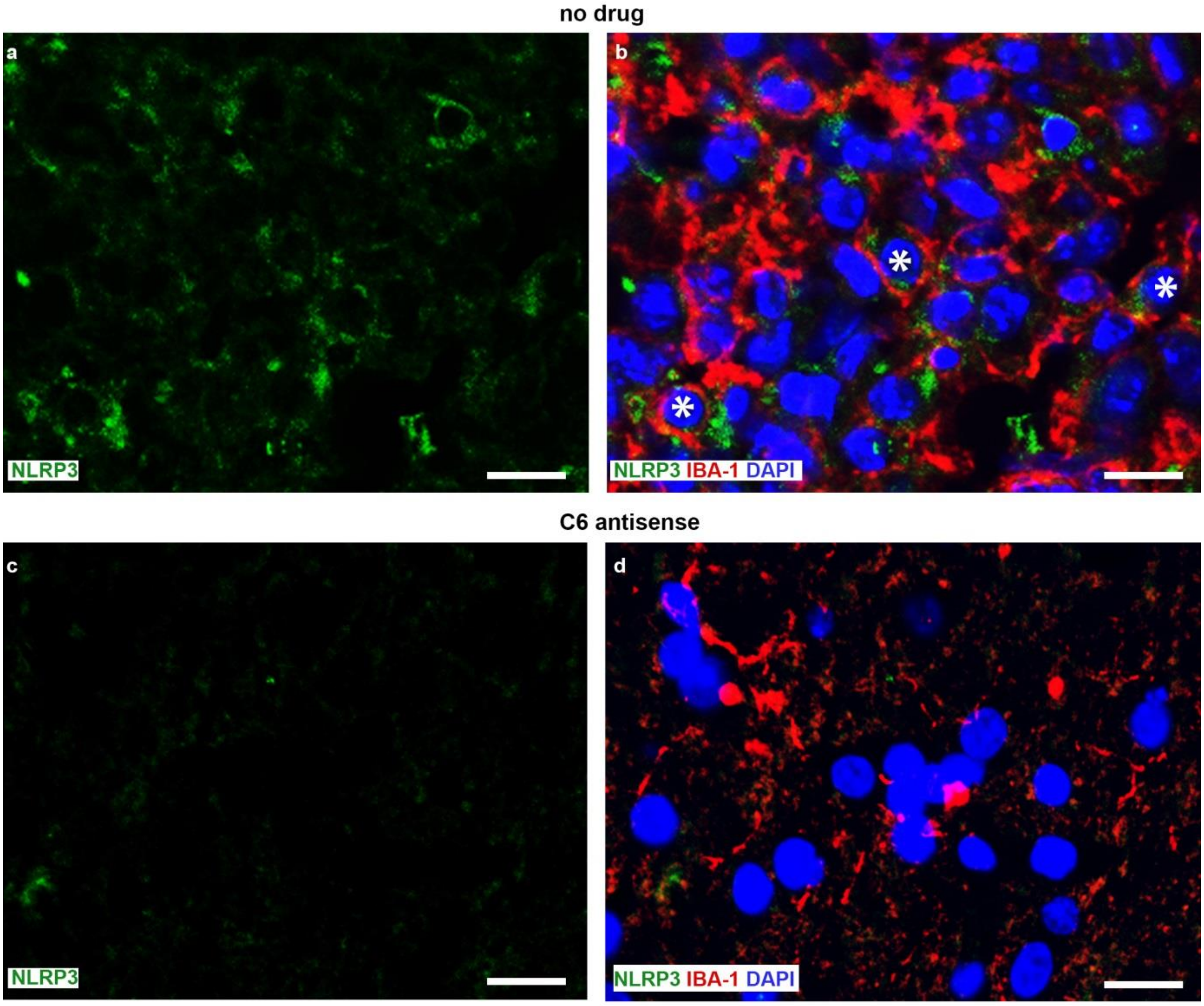

Fig. S6 Systemic administration of C6 antisense prevents NLRP3 inflammasome expression in the spinal cord of the chronic relapsing EAE model. Double immunolabelling of NLRP3 (in green), marker of inflammasome activity, and ionized calcium-binding adapter molecule 1 (IBA-1, in red) showed abundant microglia/macrophages (asterisks) and an activated NLRP3 inflammasome in the no drug mice (**a**, **b**), but on the other hand, sparse microglia/macrophages and no signs of NLRP3 expression in the C6 antisense-treated mice (**c**, **d**). Nuclei in **b** and **d** were stained with 4',6-diamidino-2-phenylindole (DAPI, blue). Scale bars: (**a**-**d**) 10 µm**.**
